# Research Progress on Genetic Basis of Fruit Quality Traits in Apple (*Malus* × *domestica*)

**DOI:** 10.3389/fpls.2022.918202

**Published:** 2022-07-14

**Authors:** Wenjun Liu, Zijing Chen, Shenghui Jiang, Yicheng Wang, Hongcheng Fang, Zongying Zhang, Xuesen Chen, Nan Wang

**Affiliations:** ^1^State Key Laboratory of Crop Biology, College of Horticulture Science and Engineering, Shandong Agricultural University, Tai’an, China; ^2^Collaborative Innovation Center of Fruit & Vegetable Quality and Efficient Production, Tai’an, China; ^3^Engineering Laboratory of Genetic Improvement of Horticultural Crops of Shandong Province, College of Horticulture, Qingdao Agricultural University, Qingdao, China; ^4^State Key Laboratory of Crop Genetics and Germplasm Enhancement, College of Horticulture, Nanjing Agricultural University, Nanjing, China; ^5^State Forestry and Grassland Administration Key Laboratory of Silviculture in Downstream Areas of the Yellow River, College of Forestry, Shandong Agricultural University, Tai’an, China

**Keywords:** apple, genetic characteristics, quality traits, QTLs, genes

## Abstract

Identifying the genetic variation characteristics of phenotypic traits is important for fruit tree breeding. During the long-term evolution of fruit trees, gene recombination and natural mutation have resulted in a high degree of heterozygosity. Apple (*Malus* × *domestica* Borkh.) shows strong ecological adaptability and is widely cultivated, and is among the most economically important fruit crops worldwide. However, the high level of heterozygosity and large genome of apple, in combination with its perennial life history and long juvenile phase, complicate investigation of the genetic basis of fruit quality traits. With continuing augmentation in the apple genomic resources available, in recent years important progress has been achieved in research on the genetic variation of fruit quality traits. This review focuses on summarizing recent genetic studies on apple fruit quality traits, including appearance, flavor, nutritional, ripening, and storage qualities. In addition, we discuss the mapping of quantitative trait loci, screening of molecular markers, and mining of major genes associated with fruit quality traits. The overall aim of this review is to provide valuable insights into the mechanisms of genetic variation and molecular breeding of important fruit quality traits in apple.

## Introduction

Apple (*Malus* × *domestica* Borkh.) shows strong ecological adaptability and is widely cultivated around the world. In 2020, the total global planting area of apple was 4.622 million ha, with total production output of 86.443 million tonnes, distributed in almost 100 countries on six continents (FAO and WFP, 2021). Consequently, apple is among the most economically important fruit crops worldwide. Most apple fruit are crisp, juicy, sweet, and delicious, and are rich in vitamins, dietary fiber, polyphenols, and mineral elements. Apple has many benefits to human health and is a favorite fruit among consumers (Eberhardt et al., 2000; Hyson, 2011; Oyenihi et al., 2022). Apple fruit quality is determined by many individual traits, most of which are quantitative traits controlled by minor polygenes or oligogenes (Conner et al., 1998; Cãtãlina et al., 2015; Zheng et al., 2020). According to the research objective, fruit quality can be divided into appearance, flavor, nutritional, storage and transportation, resistance, and processing qualities (Chen et al., 2015). The determinants of the different quality attributes include single fruit weight, fruit shape, color, texture, flavor, aroma, and functional ingredients (Chen et al., 2015).

Exploring the genetic basis and molecular mechanism of phenotypic traits is of great significance for fruit tree breeding. The inheritance of traits can be divided into qualitative traits controlled by major genes and quantitative traits controlled by minor genes. The former are inherited simply and follow Mendelian inheritance principles (Mendel, 1965), whereas the latter are affected by the environment and gene interactions, and hybrid offspring often deviate from Mendelian inheritance patterns. Over the course of long-term evolution, gene recombination and natural mutation have resulted in a high degree of heterozygosity in apple (Sun et al., 2020). In addition, owing to extensive artificial selection, non-additive genetic effects are important sources of genetic variation for apple phenotypes (Kumar et al., 2015). These genetic characteristics, in conjunction with a perennial life history and long juvenile phase, complicate research on apple genetics.

Previously, reverse genetics was used to explore the genes that influence apple fruit quality. For example, the V-myb myeloblastosis viral oncogene homolog transcription factors (TFs) *MdMYB1* and *MdMYB10* regulate anthocyanin synthesis and coloration in apple fruit (Takos et al., 2006; Espley et al., 2007). The sucrose transporter *MdSUT1* and sorbitol transporter *MdSOT1* regulate the sugar uptake and transport mechanism (Fan et al., 2009). The 1-aminocyclopropane-1-carboxylic acid synthase gene (*MdACS1*) regulates ethylene synthesis and apple fruit ripening (Sunako et al., 1999). The successful assembly of the complete genome sequence of “Golden Delicious” apple in 2010 has enabled whole-genome analysis of apple. In addition, availability of a reference genome has permitted related research on apple fruit quality, including genome association analysis, and the cloning and identification of key functional genes. A total of 57,386 genes, including 4,021 TFs, 178 microRNAs (miRNAs), 992 resistance genes, and 1,246 biosynthetic genes, were annotated in the “Golden Delicious” genome (Velasco et al., 2010). Subsequently, additional high-quality apple genome assemblies have been generated using the latest sequencing technologies or more homozygous and diverse samples (Li et al., 2016; Daccord et al., 2017; Zhang et al., 2019; Sun et al., 2020). In addition, substantial progress has been achieved in genome-resequencing analysis and genome-wide association analysis (GWAS) (Duan et al., 2017; Liao et al., 2021). These achievements are essential for the genetic analyses of apple fruit traits, for example, for the construction of high-density linkage maps, mapping of quantitative trait loci (QTLs), and identification of genes crucial for apple trait development.

This review focuses on the genetic variation characteristics of important fruit quality traits in apple. We summarize recent genetic studies on apple fruit quality traits, including appearance, flavor, nutritional, ripening, and storage qualities. In addition, we discuss the mapping of QTLs, screening of molecular markers, and mining of major genes associated with each quality trait. The overall aim of this review is to provide valuable insights into the mechanisms of genetic variation and molecular breeding of important fruit quality traits in apple.

## Apple Fruit Appearance Quality

The appearance quality of apple fruit, mainly comprising fruit size (single fruit weight), fruit shape, and fruit color, is an important aesthetic attribute that strongly determines the commercial value of the fruit. For example, the breeding of the large-fruit cultivar “World No. 1” (a single fruit weighs more than 600 g), the long-oval shaped cultivar “Starkrimson” (fruit shape index of 0.98; Westwood and Blaney, 1963), and various red-colored cultivars all represent the genetic improvement of apple fruit appearance and quality (Chen et al., 2021). Here, we present an overview of the genetic characteristics of fruit appearance quality, such as single fruit weight, fruit shape, coloration, and fruit russeting, and discuss the QTLs, molecular markers, and main-effect genes associated with each appearance quality trait.

### Single Fruit Weight

**FIGURE 1:**
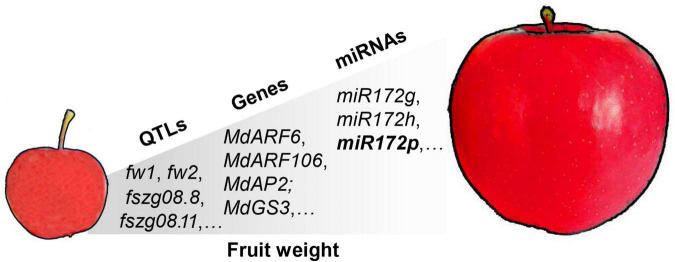
Quantitative trait loci (QTLs), molecular markers, major-effect genes, and miRNAs associated with apple fruit weight. The main QTLs associated with fruit weight include *fw1*, *fw2*, and *fszg08.8* (Devoghalaere et al., 2012; Chang et al., 2014). The major-effect genes include the auxin response factors *MdARF6* and *MdARF106*, and APETALA 2 family TF *MdAP2* (Devoghalaere et al., 2012; Yao et al., 2015). The main miRNAs include *miR172g*, *miR172h*, and *miR172p* (Yao et al., 2015; Duan et al., 2017). The function of *miR172p* has been elucidated in transgenic “Royal Gala” apple.

Fruit weight is an economically important trait in apple and is a quantitative trait controlled by multiple genes (Brown, 1960). As a quantitative indicator of apple fruit size, it directly determines the market price of the fruit. Almost 100 QTLs associated with apple fruit weight have been reported, with logarithm of the odds (LODs) ranging from 2.97 to 10.98, and include major and minor loci distributed on almost all 17 chromosomes (Liebhard et al., 2003; Kenis et al., 2008; Devoghalaere et al., 2012; Chang et al., 2014; Potts et al., 2014). Devoghalaere et al. (2012) detected six QTL regions for fruit weight. Two of the QTLs were conserved across both segregating populations on linkage group (LG) 8 (designated *fw2*) and LG 15 (designated *fw1*). One QTL was mapped to a region containing an auxin response factor gene (*ARF106*). This gene is expressed during cell division and cell expansion, consistent with a potential role in the control of fruit size (Devoghalaere et al., 2012). Consistently, transcriptome analysis of “Longfeng” apple and its large-fruited bud sport variety “Grand Longfeng” showed that most of the differential genes were related to auxin signaling, including the auxin synthetic genes *MdTAR1* and *MdYUCCA6* (Bu et al., 2020). These results suggested that auxin played a critical role in trait development of single fruit weight in apple. Recent apple genome resequencing studies identified one cell division regulatory gene and two β-galactosidase genes in *fw1* by GWAS analysis. In addition, the miRNAs *miR172g* and *miR172h* are indicated to be important for fruit enlargement during domestication of cultivated apples (Duan et al., 2017). Similarly, overexpression of *miR172p* in transgenic “Royal Gala” apple significantly reduces fruit size by targeting the expression of *AP2* TFs (Yao et al., 2015). The QTLs, molecular markers, major-effect genes, and miRNAs associated with apple fruit weight have been extensively mined, but for the majority, except *miR172p*, their functions remain to be elucidated (Figure 1).

### Fruit Color

**FIGURE 2:**
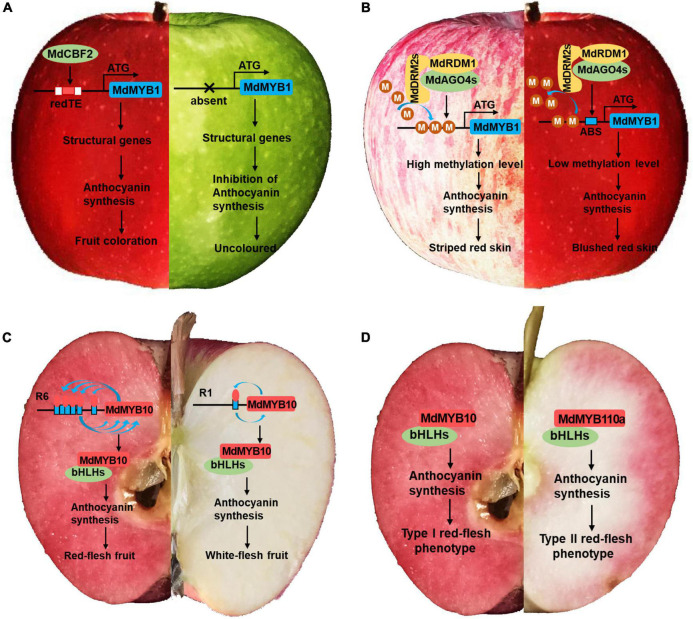
Red pigmentation of apple fruit skin and flesh. **(A)** A gypsy-like long terminal repeat retrotransposon (designated redTE) was inserted 3297 bp upstream of *MdMYB1*, thereby activating the expression of *MdMYB1* and controlling the redness of the fruit skin. **(B)** *MdAGO4s*, *MdDRM2s*, and *MdRDM1* interact with each other and form an effector complex. *MdAGO4s* recruit *MdDRM2s*, which catalyze CHH methylation of the *MdMYB1* promoter. *MdMYB1* then regulates anthocyanin accumulation to determine the coloration. M, a -CH_3_ (methyl); ABS, AGO4 binding sequence. **(C)** Model showing autoregulation of the R6 and R1 promoters by *MdMYB10*. The *MdMYB10* promoter in red-fleshed apple contains six 23 bp repeating microsatellite sequences (R6), which confer *MdMYB10* with self-activation. The *MdMYB10* promoter in white-fleshed apple contains only one 23 bp repeating microsatellite sequence. **(D)** *MdMYB10* and its homolog *MdMYB110a* are involved in the red pigmentation of type I and type II red-fleshed apples, respectively.

The fruit color of apple can be divided into skin color and flesh color. The skin color can be subdivided into presence or absence of red pigmentation, striped red, or blushed red. The flesh color is mainly subdivided into red fleshed and non-red fleshed. The red pigmentation of apple fruit is determined mainly by anthocyanins, which are color-producing secondary metabolites that accumulate in different tissues and organs of plants (Jaakola, 2013). The major gene *MdMYB1* was the first gene identified to control the presence or absence of red pigmentation in the skin (Takos et al., 2006) and associated molecular markers were explored subsequently (Kumar et al., 2012). More recently, GWAS analysis using single-nucleotide polymorphism (SNP) markers confirmed the association between fruit color and the *MdMYB1* locus on chromosome 9 (Migicovsky et al., 2016; McClure et al., 2019). Comparative genomics analysis of 148 apple populations and a segregated hybrid population revealed that a gypsy-like long terminal repeat retrotransposon (designated redTE) was inserted 3297 bp upstream of *MdMYB1*, thereby activating the expression of *MdMYB1* and controlling the redness of the skin (Figure 2A; Zhang et al., 2019). In Japanese plums, high levels of variability in the intronic and intergenic regions of the *MYB10* LG3 cluster were also closely associated with polymorphisms in their skin color (Fiol et al., 2021). These results reveal the important function of *MYB1* as a major gene in regulating fruit coloration, and its function is affected by extensive variation in gene regions and intergenic regions.

In contrast with the presence or absence of red pigmentation in the fruit ground color, the color patterns of the fruit overcolor, such as blushed red and striped red, are mostly associated with methylation modification, which represents a type of epigenetic inheritance. Differences in anthocyanin levels between the red and green stripes can be explained by differences in methylation levels of the *MdMYB10* promoter (Telias et al., 2011). Whole-genome bisulfite sequencing of “Red Delicious” and its four-generation red sport mutants “Starking Red,” “Starkrimson,” “Campbell Redchief,” and “Vallee Spur” showed that differences in DNA methylation levels were responsible for genetic variation of red sport mutants from “Red Delicious” (Li et al., 2019). Subsequent detailed methylation modification studies using striped red or blushed red fruit of “Fuji” apple as the study material showed that Argonaute 4 (*MdAGO4s*) methylated the *MdMYB1* promoter, thereby regulating anthocyanin biosynthesis (Figure 2B; Jiang et al., 2020).

Red-fleshed apples have received widespread attention from apple breeders and consumers owing to their more attractive color and higher functional nutritional content (Wang et al., 2018a). Red-fleshed apples are mainly divided into type I (red pigmentation in the fruit flesh, stems, flowers, and young leaves) and type II (red flesh only in the outer cortex, no red pigmentation in the leaves, stems, or other tissues) (Volz et al., 2009; Sekido et al., 2010). The red-flesh phenotype of type I apples is controlled by *MdMYB10*, which contains six 23 bp repeating microsatellite sequences in its promoter that confer it with self-activation, resulting in anthocyanin synthesis (Figure 2C; Espley et al., 2009). The red-flesh phenotype of type II apples is controlled by *MdMYB110*, a *MdMYB10* homolog located in LG 17, which is not expressed in type I red-fleshed fruit (Figure 2D; Chagné et al., 2013). In red-fleshed kiwifruit, both *MYB10* and *MYB110* could upregulate anthocyanin biosynthesis in fruit, while *MYB10* resulted in anthocyanin accumulation limited to the inner pericarp (Wang W. Q. et al., 2022a). These results suggest that the differential expression patterns of *MYB10* and *MYB110* contribute to the variation of the red flesh phenotype.

### Fruit Shape

Fruit shape is a quantitative trait controlled by minor polygenes. The fruit shape index is the ratio of the fruit height to width. Using multiple hybrid populations, Brown (1960) observed that the fruit shape index of most combinations was close to the parent median value. Previous studies have shown that the fruit shape index of apple is a quantitative trait controlled by five pairs of genes (Sun et al., 2012). Four simple sequence repeat markers and one amplified fragment length polymorphism marker linked to the fruit shape index were screened in a “Jonathan” × “Golden Delicious” hybrid population using bulked segregant analysis (Sun et al., 2012). Using the same hybrid population, QTL mapping analysis showed that LG11 and LG15 contained QTLs associated with fruit shape index, and the QTL loci in LG11 were stable in different years (Chang et al., 2014). Interestingly, there are not only overlapping QTLs for single-fruit weight and fruit shape, but also many independent QTLs. However, to date, the crucial genes that regulate apple fruit shape have not been identified.

### Fruit Russeting

Fruit russeting occurs in many apple cultivars, such as “Egremont Russet,” and seriously affects the appearance quality and commercial value of the fruit. Fruit russeting in apple is the result of formation of a plastic periderm following microscopic cracking of the cuticle (Khanal et al., 2013). It was initially considered that fruit russeting in apple might be controlled solely by the *Ru* gene. However, evaluation of the offspring of crosses of non-fully russeted and less-russeted cultivars with fully russeted cultivars revealed that the non-fully russeted phenotype was controlled by multiple factors (Alston and Watkins, 1973). A high-density genetic map was developed using a F_1_ segregating population derived from a cross between the fully russeted cultivar “Renetta Grigia di Torriana” and “Golden Delicious,” and a major QTL associated with fruit russeting, designated *Ru_RGT*, was detected on chromosome 12. In addition, a putative ATP-BINDING CASSETTE G family transporter *ABCG* has been identified as a candidate gene that controls russet development in apple (Falginella et al., 2015). Overexpression of *MdMYB93* in tobacco leaves leads to accumulation of a large amount of lignin monomers, suberin, and their precursors (Legay et al., 2016), suggesting that *MdMYB93* may promote the formation of apple fruit russeting. In contrast, MdLIM1 can directly bind to the CCACTTGAGTAC element in the phenylalanine ammonia-lyase promoter, thereby inhibiting lignin synthesis and reducing the formation of fruit russeting (Yuan et al., 2019). However, the functional verification of the genes regulating fruit russeting so far is only related to the lignin synthesis, and the subsequent direct relationship with the fruit russeting phenotype remains to be verified.

## Flavor and Nutritional Quality of Apple Fruit

With economic development and improvement of living standards, increasing attention is paid to the flavor quality, nutritional value, and health benefits of fruit. While consumers are influenced by aesthetic traits, they also are paying greater attention to taste attributes. Here, we introduce the genetic characteristics of apple fruit flavor and nutritional components, such as soluble sugars, organic acids, aromatic substances, and polyphenols, and discuss the QTLs, molecular markers, and main-effect genes associated with each quality trait.

### Soluble Sugars

In apple, sugar and acid contents and the sugar:acid ratio are important indicators of fruit flavor quality (Jayasena and Cameron, 2008). The soluble sugars in apple fruit mainly comprise sucrose, fructose, glucose, and sorbitol, among which glucose and fructose are almost entirely localized to vacuoles (Yamaki, 1984). The sugar content of apple fruit is a typical quantitative trait controlled by minor genes (Liao et al., 2021). The sugar content in hybrid offspring shows a normal distribution and the average sugar content in the population is close to the parent median (Ma et al., 2015), which indicates that the of sugar content is mainly controlled by additive genetic effects. More than 70 QTLs associated with fruit sugar content have been detected in different populations, distributed on at least 15 chromosomes with LODs ranging from 1.9 to 12.6 (Table 1). Taken together, the focal LGs for sugar content in apple fruit are LG1 and LG3, which have been located frequently in multiple hybrid populations.

**TABLE 1:** Linkage mapping for soluble sugar content in apple fruit.

Populations	Linkage group (LG)	Logarithm of the odds (LOD)	Markers	References
“Fiesta” × “Discovery”	LG3, LG6, LG8, LG9, LG14	1.9∼4.9	NA	Liebhard et al., 2003
“Telamon” × “Braeburn”	LG2, LG10	3.3∼12.6	EAATMCCT108 CH03d11	Kenis et al., 2008
1120 seedlings in seven full sib families	LG6	NA	ss475878574	Kumar et al., 2012
“Orin” × “Akane”	LG5, LG6, LG10, LG12, LG15, LG16	2.79∼8.26	Hi15a13, Hi09b04, CH05c06, CH05d11, CH03d07, CH05d11, MEST147, TsuENH109	Kunihisa et al., 2014
“Fuji” × “Delearly” “Fuji” × “Cripps Pink” “Golden Delicious” × “Scarlet” “Golden Delicious” × “Braeburn”	LG6, LG8 NA LG8 NA	3.39∼3.95 3.61	GDsnp01682 GDsnp00747 GDsnp00862	Costa, 2015
233 seedlings, 32 cultivars, 9 advanced selections	LG1, LG2, LG3, LG4, LG5, LG9, LG11, LG12, LG13, LG15, LG16	NA	ss475883868 ss475876959 ss475877464 ss475877847…	Guan et al., 2015
“Jonathan” × “Golden Delicious”	LG1	3.5∼4.3	huC01.18233570 emC01.11115376 huC01.18378291	Sun et al., 2015
“Fuji” × “Hongrou”	LG2	2.12	CH05d11-430m	Liu et al., 2016
“Jiguan” × “Wangshanhong”	LG3, LG4	3.41∼7.73	MdSNPui0843 MdSNPui05013	Ma et al., 2016
85 cultivars	LG8	NA	Chr8:24235959	Amyotte et al., 2017
110 cultivars	LG1, LG7, LG11	NA	Chr1:30129468 Chr1:30221387	Larsen et al., 2019
461 apple accessions	LG1, LG3, LG7, LG9, LG10, LG11	NA	Chr3_35640501 Chr10_11639616 Chr10_11639656 MdWD40, MdSOT2	Liao et al., 2021
“Honeycrisp” × “Qinguan”	LG1	4.71, 4.14	lm2151, MdSDH2	Wang et al., 2022

*NA, Not applicable.*

Whole-genome resequencing of 497 apple accessions revealed that apple breeding has resulted in a reduction in the degree of population-level genetic polymorphisms, and that sweet apples and wine apples were domesticated independently (Liao et al., 2021). GWAS for soluble sugar contents showed that a SNP mutation of the main-effect gene *MdSOT2* significantly reduced accumulation of sorbitol in apple fruit (Liao et al., 2021). In addition, a stable QTL was detected in LG01 in “Honeycrisp.” The SNP A/G variation of the sorbitol dehydrogenase gene *MdSDH2* promoter affects its binding to the TF *MdABI3*, thereby downregulating the expression level of *MdSDH2* and reducing the fructose content in the fruit (Wang Z. et al., 2022b). Thus, SNP mutations in genes that encode key enzymes for sugar synthesis have significant effects on the accumulation of various soluble sugars.

The unloading of sugars from the phloem to the fruit via the apoplast and symplast pathways, and the transport of sugars to the plasma and tonoplast membranes of fruit flesh cells are regulated by various genes, such as the sucrose transporters *MdSUT1*, *MdSUT2*, and *MdSUT4*, monosaccharide transporter *MdTMT1*, glucose transporter *MdVGT1*, and multiple *MdSWEET* genes (Fan et al., 2009; Ma et al., 2017; Zhen et al., 2018; Peng et al., 2020; Xu et al., 2020). The genetics, synthesis, metabolism, and transport of soluble sugars in apple fruit are complex and further research is needed.

### Organic Acids

The predominant organic acid in apple fruit is malic acid, which accounts for more than 80% of the total acid content and is the main contributor to tartness in the fruit (Scherer et al., 2012). The content of malic acid is jointly regulated by the major gene *Ma* and a minor gene (Verma et al., 2019). The recessive homozygous *mama* genotype exhibits low acidity, whereas the dominant homozygous *MaMa* and heterozygous *Mama* genotypes are controlled by minor polygenes and exhibit high to moderate acidity (Xu et al., 2012). Using a molecular marker linkage map, the major gene associated with malic acid was localized to LG16 and designated the *Ma* locus (Maliepaard et al., 1998). Subsequently, two QTLs associated with fruit acidity were detected, which were located in LG8 and LG16, respectively, and the QTL in LG16 was identical to that detected previously (Liebhard et al., 2003; Kumar et al., 2012; Ma et al., 2016).

On this basis, through further precise mapping, the major *Ma* locus in LG16 was localized to a 65–82 kb segment, containing 12–19 candidate genes, one of which encodes an aluminum-activated malate transporter, designated *MdALMTII* (or *Ma1*) (Bai et al., 2012; Xu et al., 2012). *Ma1* is considered to be the primary gene that determines the *Ma* locus and fruit acidity, which has been verified in a subsequent genome-wide linkage analysis (Liao et al., 2021). A SNP mutation (A/G) in the coding sequence of *Ma1* leads to premature termination of translation and reduces the malic acid content (Bai et al., 2012). The premature termination of translation changes the subcellular localization of *Ma1* and, consequently, its malate transport function is lost (Ma et al., 2015). Similarly, deletion of a coding region at the C-terminus of *MdALMT9*, a homolog of *Ma1*, leads to premature termination of its translation and reduction in malic acid content (Li et al., 2020). Therefore, SNPs or indels mutations in *Ma1* gene coding sequences significantly affect its transport function and malate content. In addition, recent studies have showed that *MdWRKY126* and *MdMa12* can lead to the accumulation of malate by regulating the activity of malate dehydrogenase (Gao et al., 2022; Zhang et al., 2022).

### Fruit Aroma

The aroma of apple fruit is composed of more than 300 different volatile substances, mainly including alcohols, aldehydes, esters, ketones, and ethers. The genetic mechanism of aroma caused by diverse volatile substances, different synthetic pathways, and multiple regulatory genes is extremely difficult to study. Except for LG8, LG10, and LG13, QTLs associated with fruit aroma may be localized to the remaining 14 LGs (Zini et al., 2005; Dunemann et al., 2009; Costa et al., 2013). Among them, LG2 and LG15 are the focal LGs for apple fruit aroma. In contrast, the main QTL associated with fruit aroma compounds in peach was detected in LG4 (Eduardo et al., 2013), and the QTL associated with γ-decalactone content in strawberry was detected in LG3 (Sánchez-Sevilla et al., 2014). Given the distinct aroma components in different fruits, the genetic linkage associations are diverse. In apple, genes have been cloned and identified for several enzymes crucial for aroma synthesis, such as *MdLOX1* encoding lipoxygenases (Schiller et al., 2015), *MdAAT1* and *MdAAT2* encoding alcohol acyltransferases (Li et al., 2006; Dunemann et al., 2012), and *MdADH1* and *MADH2* encoding alcohol dehydrogenases (Echeverrıa et al., 2004). However, the genetic mechanism and crucial regulatory genes associated with apple fruit aroma require further study.

### Nutrient Components

Apple fruit are rich in a variety of vitamins, dietary fiber, polyphenols, and mineral elements. Among these components, functional nutrients such as polyphenols, flavonoids, chlorogenic acid, and L-ascorbic acid (vitamin C) are effective in antioxidation, prevention of cardiovascular and cerebrovascular diseases, and anti-tumor effects in the human body (Eberhardt et al., 2000; Naveed et al., 2018; Bondonno et al., 2019). The polyphenols in apple fruit comprise mainly flavonoids, tannins, phenolic acids, and anthocyanins, which are quantitative traits controlled by multiple genes. Using a “Royal Gala” × “Braeburn” hybrid population, QTLs associated with the contents of polyphenols, such as chlorogenic acid, quinic acid, anthocyanin, catechin, epicatechin, quercetin, and phloridzin, were located in LG1, LG6, LG7, LG9, LG13, LG14, LG15, LG16, and LG17 with LODs ranging from 3.03 to 41.28 (Chagné et al., 2012). Among these QTLs, a crucial gene encoding leucoanthocyanidin reductase, *MdLAR1*, involved in flavonol synthesis was screened from the QTL located in LG16, and genes encoding hydroxy cinnamate transferase/hydroxy quinate transferase, *MdHCT/HQT*, involved in chlorogenic acid synthesis were screened from the QTL located in LG17 (Chagné et al., 2012). Using a “Prima” × “Fiesta” hybrid population, QTLs associated with polyphenols were located in LG1, LG8, and LG13, of which 33 metabolite quantitative trait loci (mQTLs) associated with skin phenolic compounds and 17 mQTLs associated with flesh phenolic compounds were detected in LG16 (Khan et al., 2012). Extensive progress has been achieved in understanding the genetic mechanism and regulation of polyphenol accumulation in apple fruit. TFs of the *MYB*, *bZIP*, *WRKY*, *HSF*, *ERF*, and other families are widely reported to be involved in apple polyphenol synthesis (An et al., 2017; Wang et al., 2017, 2018b,^2020^; Zhang et al., 2018). In comparison, research on other nutritional components of apple fruit has lagged behind.

L-ascorbic acid is an important antioxidant (Davey et al., 2000). The content of L-ascorbic acid in apple fruit is a quantitative trait controlled by multiple genes (Mellidou et al., 2012). Using “Telamon” × “Braeburn” hybrid populations, QTLs associated with apple fruit L-ascorbic acid were located in LG10, LG11, LG16, and LG17, among which genes for crucial enzymes, such as GDP-L-galactose phosphorylase (*GGP*) and dehydroascorbate reductase (*DHAR*), were identified. Allelic variation of *MdGGP1* and *MdGGP3* in “Braeburn” is associated with high L-ascorbic acid content in the fruit (Mellidou et al., 2012). A recent study has shown that the transcript and protein abundances of Asc Mannose pathway Regulator 1 Like 1 (*MdAMR1L1*), a regulator involved in the L-ascorbic acid synthesis pathway, are negatively correlated with L-ascorbic acid content during apple fruit development (Ma et al., 2022). Up to now, the research on apple L-ascorbic acid is still preliminary, and its regulatory mechanism and key genes still need to be further studied.

## Ripening and Storage Quality of Apple Fruit

The respiration of apple fruit is typical of a climacteric fruit. The fruit ripening and storability periods strongly determine the commercial value of different apple cultivars (Johnston et al., 2002). Inevitably, ethylene, as an important senescence-related endogenous hormone in plants, can initiate fruit ripening and synergistically complete the entire storage process, degrade cell walls, and lead to fruit softening (Giovannoni, 2004; Chaves and Mello-Farias, 2006). Here, we review the genetic characteristics of apple fruit ripening and storage quality, and discuss the relevant regulatory functions of ethylene and other factors.

### Fruit Ripening

Apple fruit ripening is a quantitative trait controlled by multiple genes, which basically conforms to the characteristics of mesotrophic variation. Using “Fiesta” × “Discovery” hybrid populations, one major-effect QTL associated with fruit ripening was located in LG3 (LOD = 4.7); the QTL originated from the early ripening parent “Discovery” and explained 16% of the phenotypic variability (Liebhard et al., 2003). Using a “Telamon” × “Braeburn” hybrid population, QTLs associated with fruit ripening were located in LG3, LG9, LG10, and LG16, with LODs ranging from 3.2 to 8.6 (Kenis et al., 2008). Through GWAS of 1168 samples of different apple genotypes and phenotypic analysis of their phenological traits, six SNPs (four on chromosome 3, one on chromosome 10, and one on chromosome 16) were retained as cofactors for ripening period at the whole-population level, which accounted for 17.2% of the phenotypic variance. Among the candidate genes, *NAC* family TFs and *AP2/ERF* family TFs were widely identified, indicating that they play key roles in apple fruit ripening (Urrestarazu et al., 2017). Consistent with this finding, the involvement of *NAC* and *AP2/ERF* family TFs in fruit ripening has been extensively demonstrated in other fruits, such as tomato, strawberry, peach, and kiwifruit (Wang et al., 2019; Gao et al., 2020; Fu et al., 2021; Kou et al., 2021; Martín-Pizarro et al., 2021).

Ethylene is an important regulator of fruit ripening, and ethylene biosynthesis, ethylene receptors, and ethylene response-related genes all affect fruit ripening (Sunako et al., 1999; Varanasi et al., 2011; Tan et al., 2013). In apple, MdSnRK2-1 can phosphorylate MdHB1 and MdHB2 to enhance their protein stability and transcriptional activity toward *MdACO1*, thereby promoting ethylene synthesis and fruit ripening (Jia et al., 2022). Besides, the TF *MdMYC2* is responsive to jasmonate treatment and directly binds to the promoters of *MdACS1*, *MdACO1*, and *MdERF3*, thereby activating the ethylene signaling pathway during apple fruit ripening (Li et al., 2017). The auxin response factor MdARF5 is responsive to naphthaleneacetic acid treatment and directly binds to the promoters of *MdERF2*, *MdACS1*, *MdASC3a*, and *MdACO1*, and thereby induces ethylene biosynthesis during apple fruit ripening (Yue et al., 2020). These findings indicate that different hormonal signals are closely associated with ethylene synthesis and fruit ripening.

### Fruit Storage

Fruit storability is a commercially important trait of apple, which directly determines the shelf life and commodity value. Fruit storability can be evaluated by retention of firmness and crispness, which are quantitative traits controlled by multiple genes (King et al., 2001; Soglio et al., 2009; Longhi et al., 2013). Ethylene plays an important role in the formation and maintenance of fruit firmness and crispness. The transcriptional activity of the ethylene synthesis gene *MdACS1-2* is greatly reduced by insertion in the promoter of a specific retrotransposon, namely a short interspersed nuclear element, resulting in significant reduction in ethylene synthesis and improved storage stability (Sunako et al., 1999). *MdACS1-2/-2* homozygous apple cultivars show lower ethylene production, higher fruit firmness during storage, and are generally storable, whereas *MdACS1-1/-2* heterozygous and *MdACS1-1/-1* homozygous cultivars show higher ethylene production, the fruit softens readily, and is generally intolerant of storage (Sunako et al., 1999; Harada et al., 2000; Oraguzie et al., 2004). Recent research has shown that genetic variation of the ethylene-responsive factors MdERF3 and MdERF118 is involved in regulating flesh firmness and crispness retention of apple fruit (Wu et al., 2021).

Differences in the activities of various cell wall metabolic enzymes, such as polygalacturonase (PG) and β-galactosidase (β-Gal), also contribute to variation in fruit firmness and crispness among apple cultivars (Nybom et al., 2020). Comparison of the *MdPG1* sequence between “Fuji” and “Mondial Gala” with significantly different storage traits revealed a SNP (T/T in “Fuji” and G/T in “Mondial Gala”) in the exon, which resulted in an amino acid change from valine to phenylalanine (Costa et al., 2010). Subsequently, 22 SNPs (10 in exons and 12 in introns) in *MdPG1* were identified by genome-whole resequencing analysis of 77 apple cultivars, of which six SNPs lead to changes of MdPG1 amino acid and polygalacturonase activity (Longhi et al., 2013). QTLs associated with fruit firmness have been detected on chromosomes 12, 16, and 17 by GWAS analysis, and three polygalacturonases, one pectinesterase, and one glucan endo-1,3-β-glucosidase have been identified that are associated with cell wall modifications (Duan et al., 2017).

## Conclusion

Genetic characteristics research is an important basis for crop breeding. This review has summarized research on the genetic variation characteristics of important quality traits in apple fruit in recent decades. Compared with annual and biennial self-compatible field or vegetable crops, perennial fruit trees mainly show the following characteristics of genetic variation: First, most perennial fruit trees are self-incompatible, highly heterozygous, and have a long juvenile phase. Consequently, it is difficult to design specific experiments to conduct genetic research on flowering and fruits. Second, most quality traits of fruit trees are quantitative traits controlled by multiple genes. This is the case for fruit quality traits such as fruit size, color, sugar and acid contents, aroma, and polyphenol content. However, in self-compatible peach genotypes, more than 20 fruit quality traits are controlled by one or two genes, such as white flesh/yellow flesh (Y/y), freestone/clingstone (F/f), and melting/non-melting (M/m) (Hesse, 1975). Finally, the prevalence of self-incompatibility and heterozygosity leads to non-additive genetic effects as an important source of genetic variation in apple phenotypes (Kumar et al., 2015).

In recent years, with the development of omics technologies, such as genomics, transcriptomics, proteomics, metabolomics, and phenomics, as well as GWAS, metabolic GWAS, structural variation analysis, molecular marker-assisted selection, and other emerging molecular technologies, substantial progress has been achieved in the genetic research of fruit traits. Many crucial genes associated with fruit quality traits have also been identified. However, owing to limitations in fruit tree gene editing and the generally longer juvenility period, the function of most fruit quality trait-related genes has not been fully validated (Lobato-Gómez et al., 2021). Furthermore, the heritable variation of the genome caused by epigenetics, such as histone modification, DNA methylation modification, chromatin remodeling, and non-coding RNA regulation, complicates the study of quality traits in fruit trees. Consequently, there remains much to do to comprehensively elucidate the genetic mechanism of fruit quality traits.

## Author Contributions

NW, WL, and ZC conceived the idea and prepared the figures. WL, ZC, SJ, YW, HF, and ZZ contributed to the writing. XC and NW reviewed and edited the manuscript. All authors have read and approved the final version of the manuscript.

## Conflict of Interest

The authors declare that the research was conducted in the absence of any commercial or financial relationships that could be construed as a potential conflict of interest.

## Publisher’s Note

All claims expressed in this article are solely those of the authors and do not necessarily represent those of their affiliated organizations, or those of the publisher, the editors and the reviewers. Any product that may be evaluated in this article, or claim that may be made by its manufacturer, is not guaranteed or endorsed by the publisher.
